# SEC61G overexpression and DNA amplification correlates with prognosis and immune cell infiltration in head and neck squamous cell carcinoma

**DOI:** 10.1002/cam4.4301

**Published:** 2021-09-30

**Authors:** Tianzhu Lu, Yiping Chen, Xiaochang Gong, Qiaojuan Guo, Canyang Lin, Qingfeng Luo, Ziwei Tu, Jianji Pan, Jingao Li

**Affiliations:** ^1^ Department of Radiation Oncology Jiangxi Cancer Hospital of Nanchang University Nanchang Jiangxi China; ^2^ NHC Key Laboratory of Personalized Diagnosis and Treatment of Nasopharyngeal Carcinoma (Jiangxi Cancer Hospital of Nanchang University) Nanchang Jiangxi China; ^3^ Jiangxi Key Laboratory of Translational Cancer Research Jiangxi Cancer Hospital of Nanchang University Nanchang Jiangxi China; ^4^ Department of Radiation Oncology Fujian Cancer Hospital & Fujian Medical University Cancer Hospital Fuzhou Fujian China; ^5^ Fujian Provincial Key Laboratory of Translational Cancer Medicine Fujian Cancer Hospital Fuzhou Fujian China; ^6^ Department of Pathology Jiangxi Cancer Hospital of Nanchang University Nanchang Jiangxi China

**Keywords:** biomarker, copy number variation, head and neck squamous cell carcinoma, immune cell infiltration, SEC61G

## Abstract

**Background:**

The SEC61 translocon gamma subunit (SEC61G) is a component of the SEC61 complex, which import protein into the endoplasmic reticulum. However, the correlation between SEC61G and disease prognosis in head and neck squamous cell carcinoma (HNSCC) remains unclear.

**Methods:**

SEC61G expression was analyzed using publicly available datasets. The association between SEC61G and disease prognosis was evaluated. SEC61G methylation and copy number variation were investigated and gene set enrichment analysis and gene ontology analyses identified SEC61G‐associated functions. We also investigated the correlation between SEC61G and immune cell infiltration. Finally, immunohistochemistry was used to detect SEC61G expression in oropharyngeal carcinoma.

**Results:**

SEC61G was overexpressed in pan‐cancers, including HNSCC, and negatively correlated with overall survival (OS) (*p* < 0.001 for TCGA‐HNSCC and *p* = 0.019 for GSE65858). Moreover, SEC61G was an independent prognostic factor for OS in TCGA and GSE65858 [hazard ratio (HR) = 1.80, 95% CI: 1.35–2.39, *p* < 0.001; HR = 1.87, 95% CI: 1.14–3.07, *p* = 0.013, respectively). SEC61G DNA amplification (9.66% of patients) was significantly associated with poor OS (*p* = 0.034). SEC61G overexpression and DNA amplification negatively correlated with B cell (*p* < 0.001), CD8^+^ T cell (*p* < 0.001), CD4^+^ T cell (*p* < 0.001), macrophage (*p* < 0.05), neutrophil (*p* < 0.001), and dendritic cell infiltration (*p* < 0.001). Among patients with metastatic urothelial cancer received atezolizumab, patients with high SEC61G expression had an inferior OS (*p* = 0.006). Furthermore, SEC61G protein expression was also an independent prognostic factor of OS (HR = 2.46, 95% CI: 1.15–5.28, *p* = 0.021) and progression‐free survival (HR = 2.82, 95% CI: 1.36–5.85, *p* = 0.005) for oropharyngeal cancer.

**Conclusions:**

SEC61G is overexpressed in HNSCC and is an independent prognostic factor for OS. SEC61G DNA amplification contributes to overexpression and poor outcome. Interestingly, SEC61G correlates with immune cell infiltration in HNSCC. These findings suggest that SEC61G is a potential broad‐spectrum biomarker for prognosis in HNSCC.

## Graphical abstract

SEC61G expression in oropharyngeal cancer tissues was higher than in normal adjacent tissues. Kaplan–Meier survival analyses showed that patients with high SEC61G expression had a lower overall survival and progression‐free survival than did patients with low SEC61G expression.

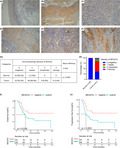

## 1 INTRODUCTION

Head and neck squamous cell carcinoma (HNSCC) is a common malignancy that accounts for 500,000 new cases every year globally.^1^, ^2^ The occurrence of HNSCC associates closely with cigarette use, alcohol use, and virus infection.^3^ Because most HNSCC patients are diagnosed in later stages, almost half of patients will experience recurrence within 3 years, and the 5‐year overall survival (OS) rate is only about 50%.^4^, ^5^, ^6^ Tumor‐node‐metastasis (TNM) classification, which considers the tumor size, location, and metastatic state, is used to develop treatment strategies and evaluate HNSCC prognosis.^7^ However, this system is not sufficient to direct all clinical treatments and predict every prognosis, because patients with the same TNM stage and treatment may have different clinical outcomes.^8^ Therefore, it is essential to find stable and reliable tumor biomarkers to identify patients with poor prognosis and to inform more aggressive treatments. Heterogeneity is a basic HNSCC characteristic resulting from the varied epithelial origin from the upper respiratory/digestive tract, including the oral cavity, oropharynx, and hypopharynx.^9^, ^10^ Furthermore, some HNSCC tumors, such as human papillomavirus (HPV)‐associated oropharyngeal cancer, which are closely related to HPV inflection, can have a significantly better prognosis.^9^, ^11^ Because of this heterogeneity, identifying stable broad‐spectrum biomarkers are difficult.

SEC61G, also known as Sec61 translocon gamma subunit, is a component of SEC61, a heterotrimeric channel protein composed of the SEC61 α, β, and γ subunits.^12^ The SEC61 complex forms a transmembrane pore and transports nascent polypeptides and proteins to the ER, thereby mediating membrane protein degradation.^13^, ^14^ Interestingly, SEC61G is upregulated in glioblastoma multiforme^15^ and gastric cancer.^16^ Previous studies show that SEC61G is necessary for tumor cell survival and cellular responses to endoplasmic reticulum stress.^17^ Knocking out SEC61γ expression triggers apoptosis, blocks EGFR/AKT survival signaling,^17^ and inhibits tumor cell growth. A recent study indicates that SEC61G overexpression was an inferior prognostic factor in glioblastoma multiforme.^15^ However, the expression and significance of SEC61G in HNSCC remain unclear.

In this study, we comprehensively evaluated the prognostic value of SEC61G expression in HNSCC patients from the Cancer Genome Atlas (TCGA) and validated the associated prognostic value in HNSCC cases from the Gene Expression Omnibus (GEO) databases. We also analyzed the effects of methylation and copy number variation (CNV) on SEC61G gene expression. Furthermore, we performed gene set enrichment analysis (GSEA) and gene ontology (GO) analyses to gain further insight into the biological role of SEC61G in HNSCC. Tumor Immune Estimation Resource (TIMER) software was used to explore the effects of SEC61G gene expression and CNV on immune infiltration. Finally, we validated SEC61G overexpression and its prognostic value in 91 oropharyngeal cancers (OPCs).

## 2 METHODS

### 2.1 Data availability

SEC61G expression in various cancer types was identified in the Oncomine database (https://www.oncomine.org/resource/login.html). The inclusion threshold was determined according to the following values: *p* < 0.001, |log_2_fold change| > 1.5, and gene ranking. In addition, the expression level of SEC61G in four HNSCC datasets from the Oncomine database (Pyeon Multi‐Cancer,^18^ Estilo Head‐Neck,^19^ Ye Head‐Neck,^19^ Peng Head‐Neck^20^) was included in this analyses.

Two datasets from the TCGA database (https://tcgadata.nci.nih.gov/tcga/) were included: RNA‐seq transcriptomic data and the corresponding patient clinical data from HNSCC samples. We downloaded RNA‐seq data from 528 HNSCC patients and 44 normal patients (https://cancergenome.nih.govin2018). The RNA‐seq data and the patient clinical information (Workflow Type: HTSeq‐FPKM) were acquired using TCGAbiolinks. Cases with insufficient or missing data (*patient's age, gender, clinical stage, T stage, N stage, pathological type, and HPV status*) were removed from subsequent data processing. The included data are shown in Table 1. HNSCC patients were classified into low and high SEC61G expression groups according to their median SEC61G expression value. SEC61G expression data and clinical data from dataset GSE65858 were also downloaded from the GEO database and used to validate the survival analyses. IMvigor 210 data and clinical information were obtained from the IMvigor210CoreBiologies R package.^21^

**TABLE 1:** Demographic and clinical characteristics of HNSCC patients in TCGA and GSE65858

Characteristics	TCGA	GSE65858
	*n* (%)	*n* (%)
Gender		
Female	126 (27.7)	43 (17.4)
Male	329 (72.3)	223 (82.6)
Age (year)		
<60	216 (47.5)	153 (56.7)
≥60	329 (52.5)	117 (43.3)
HPV		
Negative	378 (83.1)	197 (73.0)
Positive	77 (16.9)	73 (27.0)
T classification		
T1	30 (6.6)	35 (13.0)
T2	128 (28.1)	80 (29.6)
T3	127 (27.9)	58 (21.5)
T4	170 (37.4)	97 (35.9)
N classification		
N0	227 (49.9)	94 (34.8)
N1	80 (17.6)	32 (11.9)
N2	141 (31.0)	132 (48.9)
N3	7 (1.5)	12 (4.4)
Clinical stage		
Ⅰ	17 (3.7)	18 (6.7)
Ⅱ	85 (18.7)	37 (13.7)
Ⅲ	99 (21.8)	37 (13.7)
Ⅳ	254 (55.8)	178 (65.9)
Histologic grade		
G1	52 (11.4)	
G2	286 (62.9)	
G3	117 (25.7)	

### 2.2 Analysis of SEC61G methylation, copy number variation, and prognosis

SEC61G methylation and CNV data were obtained through the cBioPortal web platform (https://www.cbioportal.org/). The correlation between SEC61G methylation level and SEC61G gene expression and varying SEC61G expression in different SEC61G CNV groups were conducted. The UALCAN online tool (http://ualcan.path.uab.edu/) was used to analyze the SEC61G expression between HNSCC and normal tissues from TCGA data. The cBioPortal web platform (https://biit.cs.ut.ee/methsurv/) was used to analyze the prognostic value of SEC61G DNA amplification in TCGA‐HNSCC cases.

### 2.3 Gene set enrichment analysis

GSEA is an analytical method that determines whether a previously defined set of genes shows statistically significant, concordant differences between two phenotypes.^22^ In this study, GSEA was carried out using the R package clusterProfiler (3.8.0)^23^ to elucidate the significant function and pathway differences between the high‐ and low‐SEC61G groups. Gene set permutations were performed 1000 times for each analysis. SEC61G expression was used as a phenotype label. The “c2.cp.kegg.v6.0.symbols.gmt” file from the MSigDB collections was chosen as the reference gene collection. The parameters were set as follows: adjusted *p*‐value <0.05, false discovery rate (FDR) < 0.25, and normalized enrichment score (|NES|) > 1.

### 2.4 Gene ontology enrichment analysis between amplified and non‐amplified SEC61G in HNSCC patients

The differentially expressed genes in the DNA amplification and non‐amplification groups were obtained using the cBioPortal online platform. The differential genes were identified by *q*‐value < 0.05 (derived from the Benjamini–Hochberg procedure) between the DNA amplification and non‐DNA amplification groups. Metascape (https://metascape.org) is a tool for gene annotation and pathway analyses,^24^ and was used to analyze the enrichment of SEC61G DNA amplification‐related DEGs by process and pathway. The GO terms for biological process, cellular component, and molecular function categories were analyzed using the Metascape online tool. Only terms reaching *p*‐value <0.01, a minimum gene count = 3, and an enrichment factor > 1.5 were considered to be significant.

### 2.5 Correlation analysis of SEC61G expression and CNV with immune cell infiltration

TIMER software^25^ was used to explore the correlation between SEC61G expression, CNV, and immune cell infiltration in HNSCC samples from the TCGA database.

### 2.6 Immunohistochemistry

Tissue samples from 91 OPC cases with diagnosed pathology treated at the Fujian Cancer Hospital from 2008 to 2017 were included in the analysis. Fifty‐six adjacent normal tissue samples were also included. This study was approved by the Hospital Review Board of Fujian Cancer Hospital, Fujian, China. The clinicopathological OPC features are shown in Table 3. The samples were fixed in formaldehyde and processed with heat‐mediated antigen retrieval in citrate buffer (PH = 6). The samples were then blocked and incubated with rabbit polyclonal anti‐SEC61G (1:50, DF12136, Affinity Biosciences) at 4°C overnight. Elivision^TM^ plus Polyer HP (Mouse/Rabbit) Immunohistochemistry (IHC) Kit (Cat. KIT‐9901, MXB biotechnologies) was used in IHC detection. Two independent pathologists, who were blinded to the clinical outcome, evaluated staining intensity.

### 2.7 Statistical analyses

The Wilcoxon rank‐sum test and Wilcoxon signed‐rank test were used to analyze SEC61G expression in non‐paired and paired samples, respectively. The Kruskal–Wallis test, Wilcoxon signed‐rank test, and Chi‐squared test were used to analyzing the relationship between clinicopathological features and SEC61G expression. Survival curves were drawn using the Kaplan–Meier method, and the differences between groups were assessed via the log‐rank test or Breslow test. OS was defined as the diagnostic data to date of death from any cause, or last follow‐up. Progression‐free survival (PFS) was defined as the diagnostic data to date of disease progression, death or last follow‐up. Multivariate analyses (MVA) using Cox proportional hazard modeling were performed to estimate the risk of death. Potential confounders included gender, age, clinical stage, and treatment. *p* < 0.05 (two‐sided) was considered statistically significant. Statistical analyses were carried out using R (version 3.6.1) and SPSS (version 24.0).

## 3 RESULTS

### 3.1 SEC61G is overexpressed in head and neck squamous cell carcinoma

**FIGURE 1:**
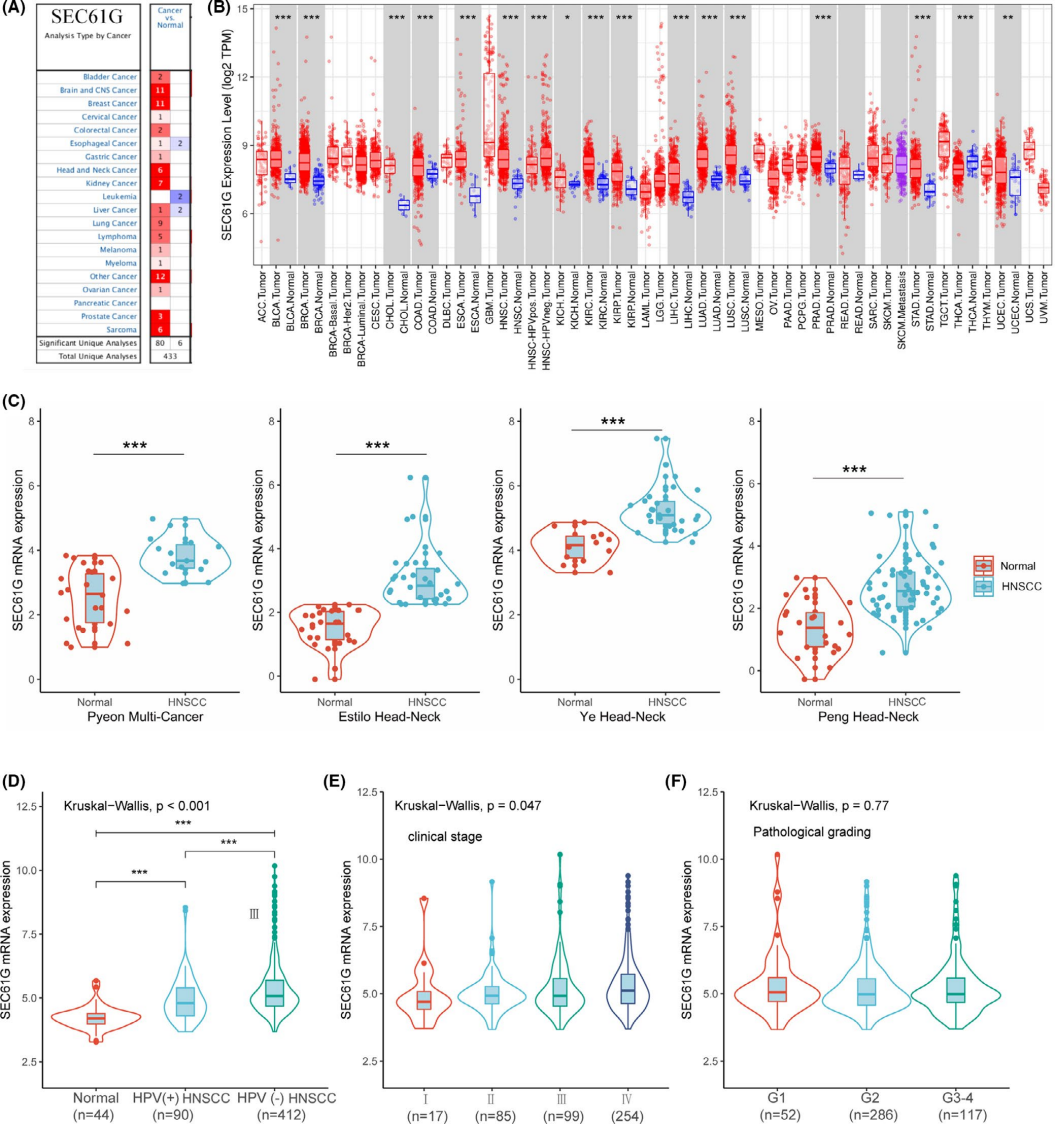
SEC61G expression levels in HNSCC and other types of human cancers. (A) SEC61G expression levels in pan‐cancer from Oncomine database; (B) SEC61G expression levels in pan‐cancer in TCGA analyzed on TIMER software; (C) SEC61G expression levels in four GEO‐HNSCC datasets (Pyeon Multi‐Cancer, Estilo Head‐Neck, Ye Head‐Neck, Peng Head‐Neck); (D) the expression differences of SEC61G in normal tissue, HPV (+) HNSCC and HPV (−) HNSCC; (E) SEC61G expression levels between different clinical stages; (F) SEC61G expression levels between different pathological grades; (*p < 0.05, **p < 0.01, ***p < 0.001)

Using Oncomine data, we observed that SEC61G was upregulated in almost all cancer types, compared to normal tissues (*p* < 0.001, |log_2_ fold change| > 1.5 in all gene ranks), including breast cancer, kidney cancer, brain and central nervous system cancer, and head and neck cancer (Figure 1A). Then, we explored SEC61G expression in the TCGA dataset using TIMER. SEC61G was highly expressed in pan‐cancers compared to normal tissues, including HNSCC (Figure 1B). Further, SEC61G expression in HPV‐negative HNSCC was higher than expression in HPV‐positive HNSCC (Figure 1B). The analyses of the four Oncomine datasets (Pyeon Multi‐Cancer, Estilo Head‐Neck, Ye Head‐Neck, and Peng Head‐Neck) also showed that SEC61G were overexpressed in HNSCC (Figure 1C).

Furthermore, the TCGA dataset analysis showed that regardless of the HPV infection status, the expression of SEC61G was higher in HNSCC than in normal tissues (*p* < 0.001, Kruskal–Wallis test). Our results also showed the SEC61G expression correlated positively with the clinical stage (*p* = 0.047) (Figure 1D), where later disease‐stage patients tended to express more SEC61G (Figure 1E). In contrast, SEC61G expression did not correlate with pathological grade (*p* = 0.77) (Figure 1F).

### 3.2 SEC61G overexpression correlates with poor overall survival in HNSCC

**FIGURE 2:**
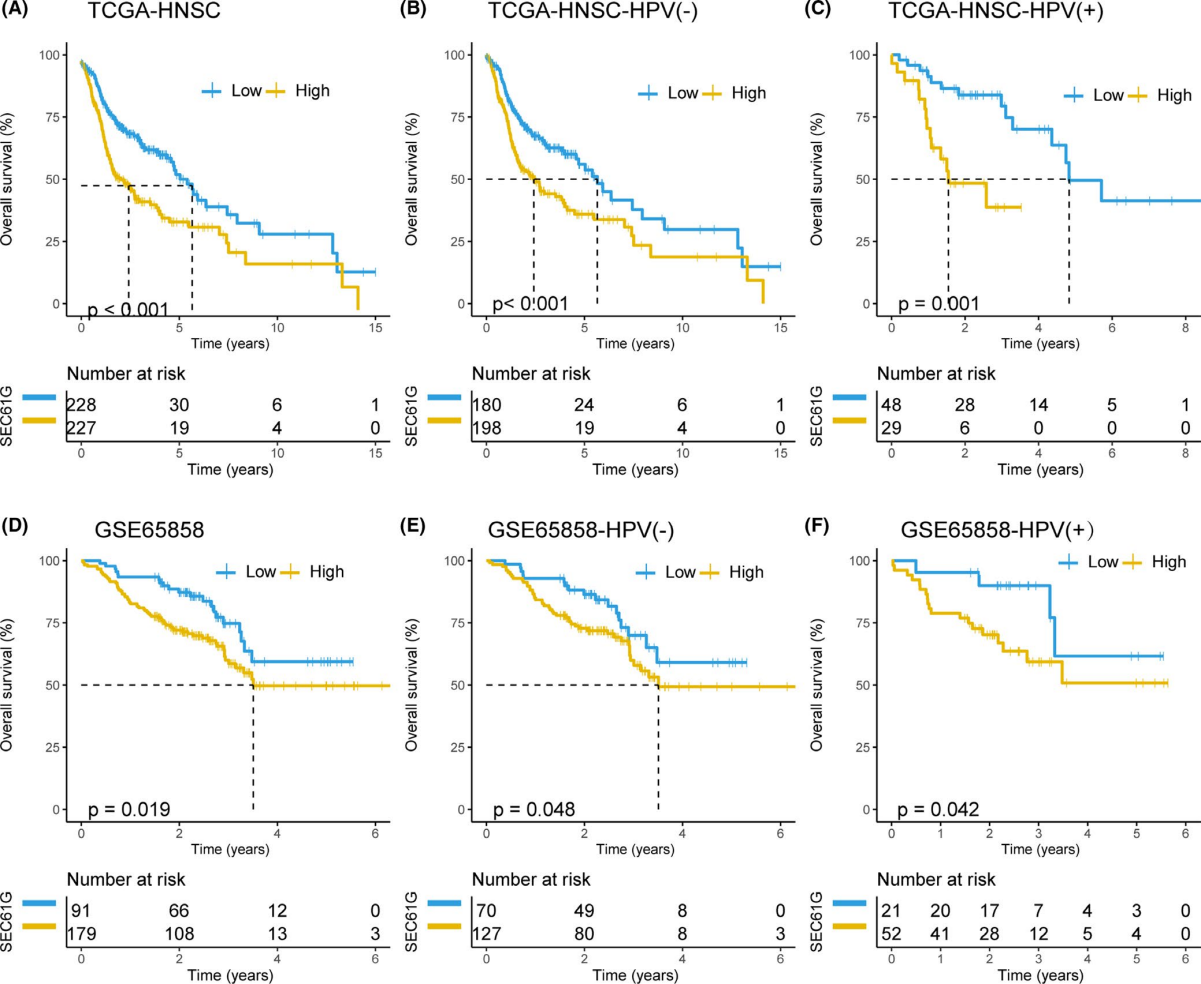
The prognostic values of SEC61G expression in HNSCC. (A) Overall survival curve of SEC61G in TCGA‐HNSCC (*n* = 445); (B) Overall survival curve of SEC61G in TCGA‐HPV (−) HNSCC (*n* = 378); (C) Overall survival curve of SEC61G in HPV (+) HNSCC from TCGA (*n* = 77); (D) Overall survival curve of SEC61G from GSE65858 (*n* = 270); (E) Overall survival curve of SEC61G in HPV(−) HNSCC from GSE65858 (*n* = 197); (F) Overall survival curve of SEC61G in HPV (+) HNSCC from GSE65858 (*n* = 73)

To examine the relationship between SEC61G expression and OS, we divided patients into high‐ and low‐expression groups based on the median SEC61G expression (5.01 FPKM) in HNSCC‐TCGA. Kaplan–Meier survival analysis showed that the OS of high SEC61G‐expressing patients was significantly poorer than that of patients with low‐SEC61G expression (*p* < 0.001) (Figure 2A). Similar results were observed in HPV‐negative and HPV‐positive subgroups (*p* < 0.001 and *p* = 0.001, respectively) (Figure 2B,C). Multivariate analysis also showed that SEC61G expression was an independent prognostic factor for HNSCC [hazard ratio (HR) = 1.80, 95% CI: 1.35–2.39, *p* < 0.001] (Table 2).

**TABLE 2:** The multivariate analysis of overall survival according to SEC61G expression, after adjusting for other potential predictors in TCGA and GSE65858

Characteristics	TCGA		GSE65858	
	HR (95% CI)	*p*‐value	HR (95% CI)	*p*‐value
Gender (Female vs. Male)	0.81 (0.59–1.09)	0.163	1.01 (0.58–1.74)	0.990
Age (<60 years vs. ≥60 years)	1.14 (0.86–1.53)	0.362	1.31 (0.86–2.01)	0.214
HPV (Negative vs. Positive)	0.93 (0.61–1.41)	0.744	0.47 (0.28–0.83)	0.009
Histologic grade				
G1 versus G2	1.86 (1.14–3.03)	0.013		
G1 versus G3	1.56 (0.92–2.63)	0.100		
Clinical stage (I–II vs. III–IV)	1.24 (0.87–2.63)	0.231	1.78 (1.30–2.44)	<0.001
SEC61G (Low vs. High)	1.80 (1.35–2.39)	<0.001	1.87 (1.14–3.07)	0.013

To further verify the prognostic value of SEC61G expression in HNSCC, we analyzed the GSE65858 dataset (including survival data). Detailed clinicopathologic features are listed in Table 1. Kaplan–Meier survival analysis indicated that patients with high SEC61G expression had an inferior OS than did patients with low‐SEC61G expression (*p* = 0.019). Subgroup analyses also showed that patients with high SEC61G expression had a worse OS than did patients with low‐SEC61G expression in the HPV‐negative and ‐positive subgroups (*p* = 0.048 and *p* = 0.042, respectively) (Figure 2D–F). The MAV confirmed that SEC61G was an independent prognostic factor for OS in HNSCC (HR = 1.87, 95% CI: 1.14–3.07, *p* = 0.013) (Table 2).

### 3.3 SEC61G demethylation and DNA amplification in HNSCC

**FIGURE 3:**
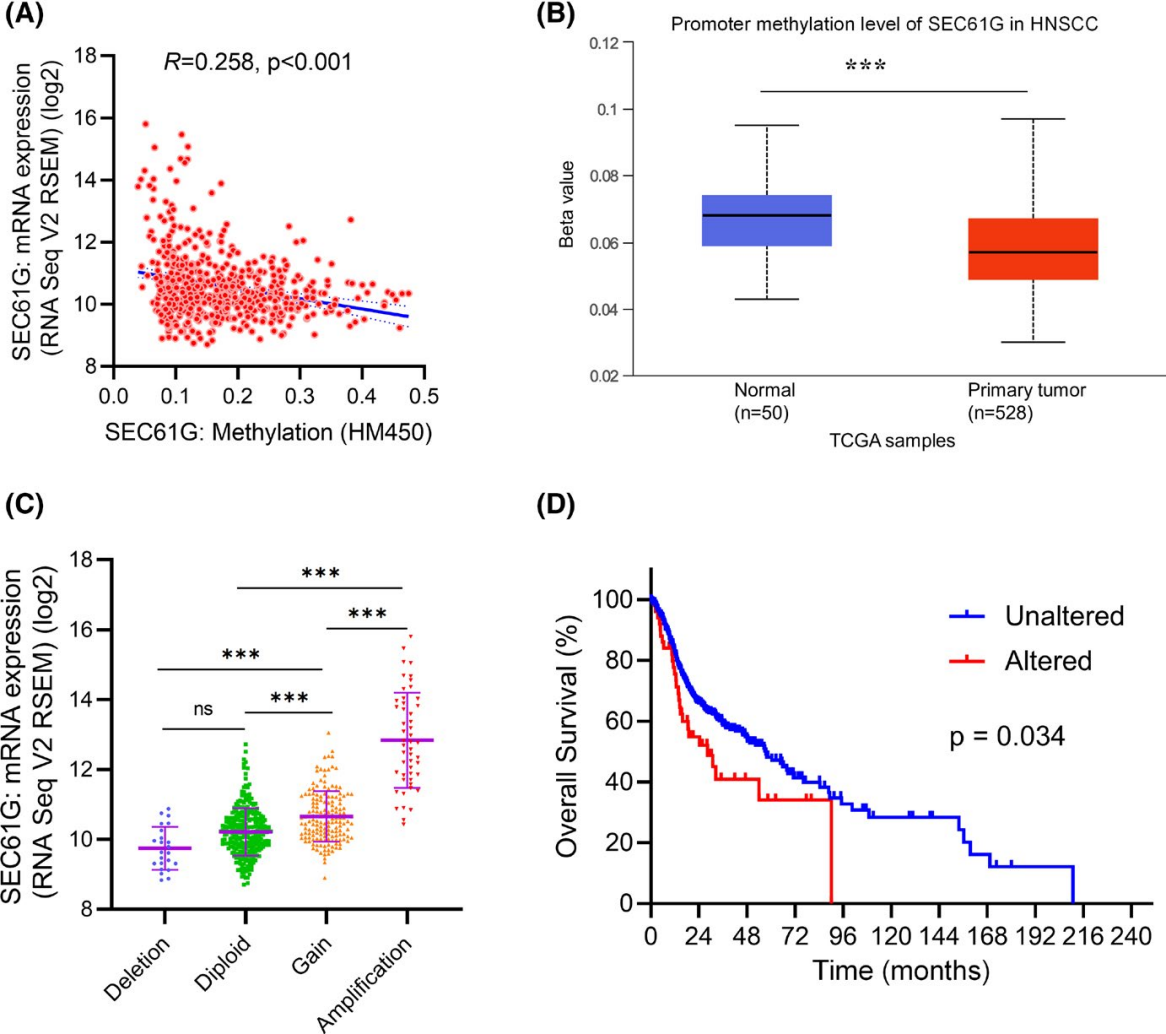
The methylation and copy number variations of SEC61G in HNSCC. (A) the correlation between SEC61G methylation and its expression level; (B) the promoter methylation of SEC61G in tumor tissues from TCGA‐HNSCC data; (C) the expression levels in different CNV of SEC61G; (D) Overall survival curve of patients in SEC61G DNA amplification (Altered) or non‐amplification (Unaltered) group. (*p < 0.05, **p < 0.01, ***p < 0.001)

To investigate the mechanism of SEC61G upregulation in HNSCC, we analyzed SEC61G methylation and CNV using cBioPortal and the UALCAN web platform. The results showed that SEC61G expression was negatively correlated with methylation (*R* = 0.258, *p* < 0.001) in HNSCC (Figure 3A). SEC61G promoter methylation in tumor tissues from the TCGA‐HNSCC dataset was significantly lower than methylation in normal tissues adjacent to tumors (*p* < 0.001) (Figure 3B).

CNV data showed that SEC61G DNA amplification was present in 9.66% (51/528) patients. Further, SEC61G expression in the DNA amplification group was significantly higher than in the other groups (Deletion, Diploid, and Gain groups) (*p* < 0.001) (Figure 3C). After grouping patients with amplified SEC61G into the altered group and the other patients into the unaltered group, Kaplan–Meier analysis showed that the OS of the altered group was lower than the OS of the unaltered group (*p* = 0.034) (Figure 3D).

### 3.4 Functional enrichment analyses by GSEA and GO

**FIGURE 4:**
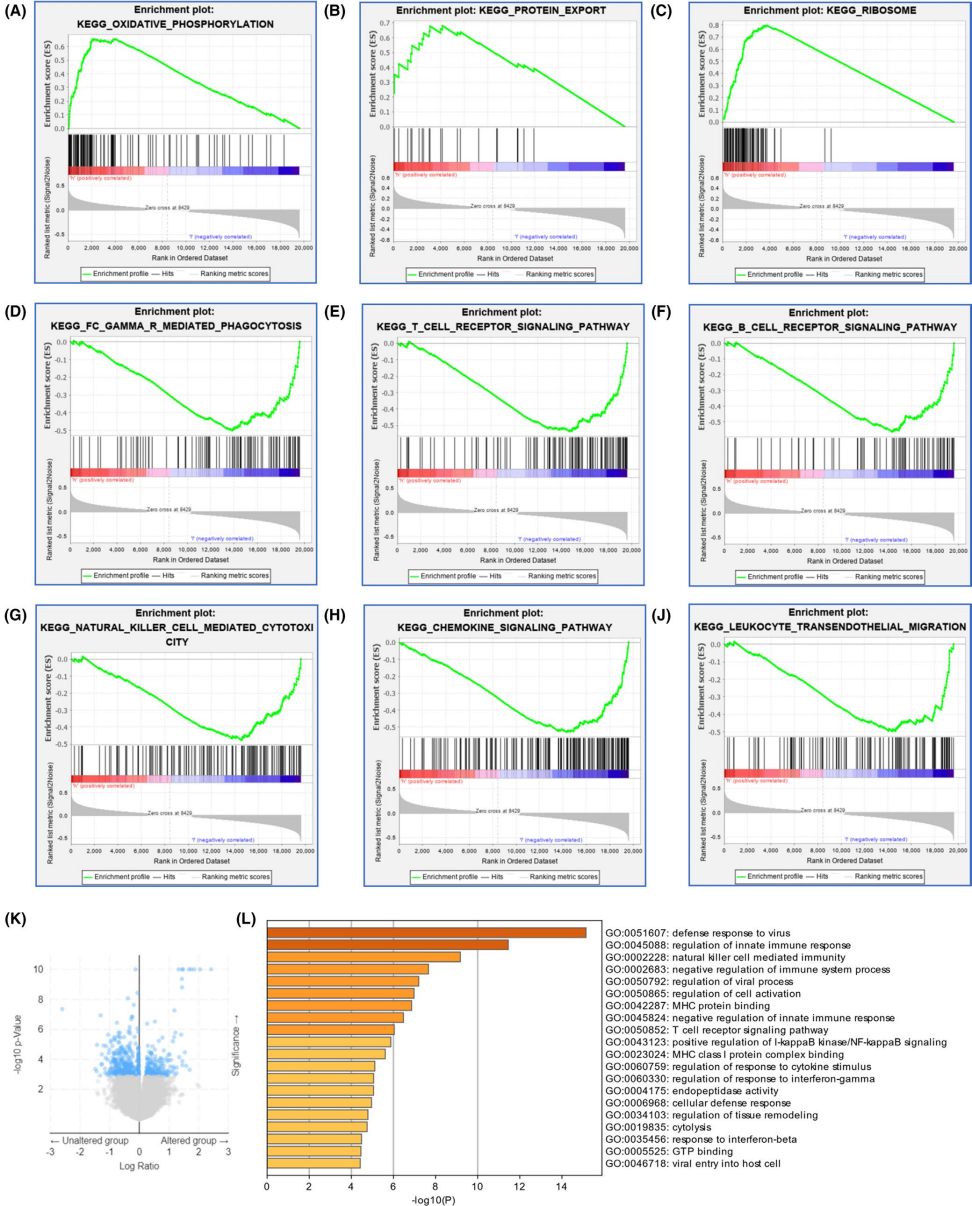
Functional enrichments of SEC61G in HNSCC. (A–K) Functional enrichments by GSEA: (A) Enrichment of genes in the oxidative‐phosphorylation;(B) Enrichment of genes in the protein export; (C) Enrichment of genes in proteasomes; (D) Enrichment of genes in Fc‐gamma R‐mediated phagocytosis; (E) Enrichment of genes in T‐cell receptor signaling pathway; (F) Enrichment of genes in the B‐cell receptor signaling pathway; (G) Enrichment of genes in the nature killer cell‐mediated cytotoxicity; (H) Enrichment of genes in the chemokine signaling pathway; (I) Enrichment of genes in the leukocyte trans‐endothelial migration; (J) Volcano plot of differentially expressed genes (DEGs) between altered (DNA amplification) group and unaltered (non‐amplification) group; (K) GO enrichment analysis of differentially expressed genes (DEGs) in altered group and unaltered group

To explore the potential biological functions of SEC61G that promote tumor progression, we divided patients into high‐ and low‐expression groups based on the median SEC61G expression. GSEA analyses showed that high SEC61G expression positively upregulated the signal pathways involving oxidative‐phosphorylation, protein export, and proteasomes (Figure 4A–C). However, Fc‐gamma R‐mediated phagocytosis, T‐cell receptor signaling pathway, B‐cell receptor signaling pathway, natural killer cell‐mediated cytotoxicity, chemokine signaling pathway, and leukocyte trans‐endothelial migration were downregulated (Figure 4D–I).

To further elucidate the biological functions of SEC61G, we analyzed the differentially expressed genes (DEGs) between the DNA amplification and non‐amplification groups (Figure 4J). GO analysis showed that 16 biological processes (BP) and 4 molecular functions (MF) were enriched (Figure 4K). Among the 16 BP terms, 10 were associated with immune responses, including “regulation of innate immune response,” “negative regulation of immune system process,” “natural killer cell‐mediated immunity,” “regulation of cell activation,” “negative regulation of innate immune response,” “T cell receptor signaling pathway,” “positive regulation of IκB kinase/NF‐κB signaling,” “regulation of response to cytokine stimulus,” “regulation of response to interferon‐gamma,” and “response to interferon‐beta.” The remaining BP terms were “defense response to virus,” “regulation of viral process,” “cellular defense response,” “regulation of tissue remodeling,” “cytolysis,” and “viral entry into host cell.” The 4 MF terms were “MHC protein binding,” “MHC class I protein complex binding,” “endopeptidase activity,” and “GTP binding” (Figure 4K).

### 3.5 SEC61G expression negatively correlates with immune infiltration in HNSCC

**FIGURE 5:**
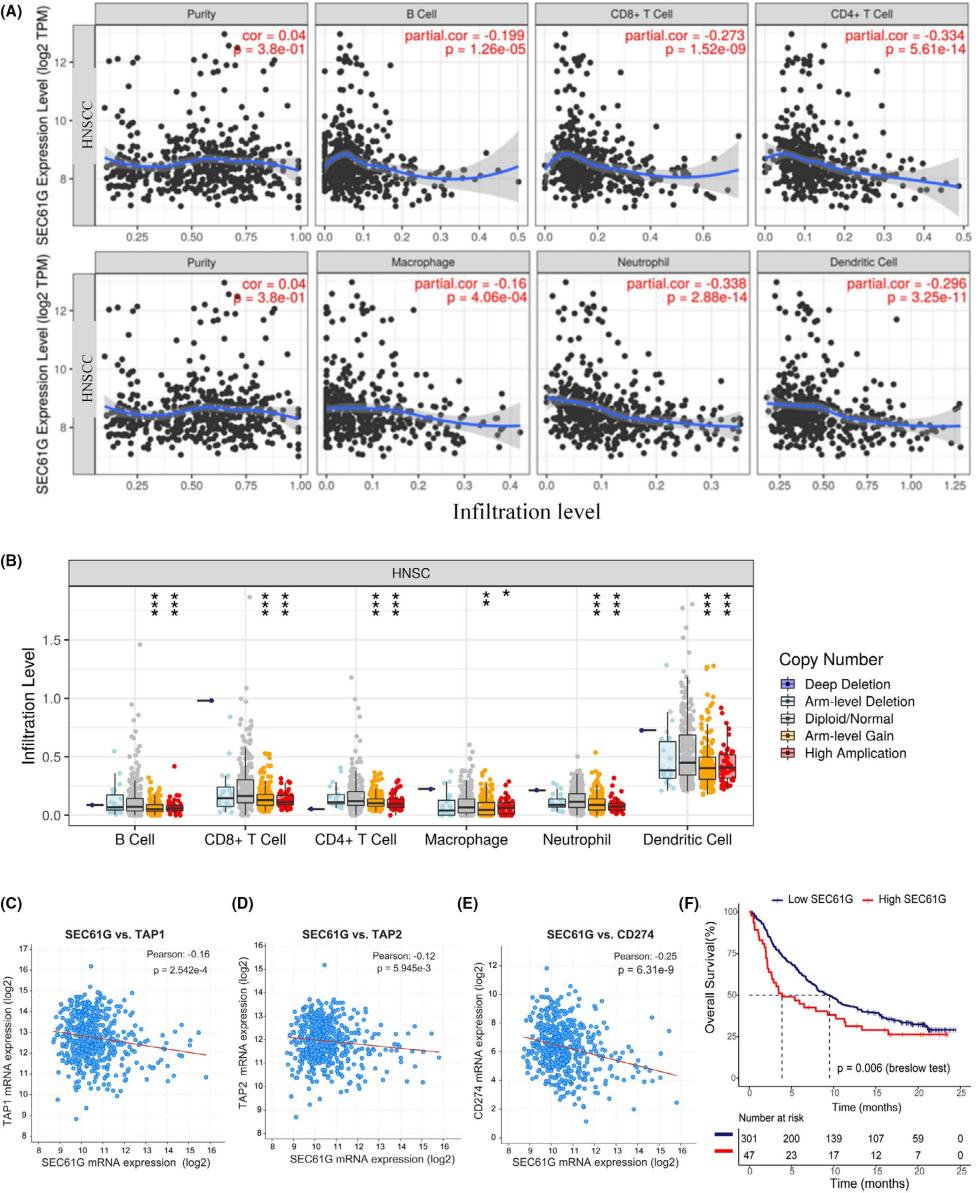
The correlation of SEC61G expression and copy number variation with immune infiltrations in HNSCC. (A) The correlation between the infiltrations of immune cells and the expression of SEC61G via TIMER (Spearman's correlation); (B) The correlation between the infiltrations of immune cells and CNVs of SEC61G via TIMER (Wilcoxon rank‐sum test); (C) The negatively expressing correlation between SEC61G and TAP1 (Pearson correlation); (D) The negatively expressing correlation between SEC61G and TAP2 (Pearson correlation); (E) The negatively expressing correlation between SEC61G and CD274 (PD‐L1) (Pearson correlation); (F) Overall survival curve of SEC61G high‐ and low‐expression groups in metastatic urothelial cancer patients with atezolizumab; (*p < 0.05, **p < 0.01, ***p < 0.001)

Considering that the GSEA and GO analyses indicated that genes and terms associated with the immune system were enriched in HNSCC, we further analyzed the correlation between SEC61G expression, CNV, and immune cell infiltration in HNSCC. We observed that SEC61G expression negatively correlated with B cell (*p* < 0.001), CD8^+^ T cell (*p* < 0.001), CD4^+^ T cell (*p* < 0.001), macrophage (*p* < 0.001), neutrophil (*p* < 0.001), and dendritic cell infiltration (*p* < 0.001) (Figure 5A). SEC61G DNA amplification was also significantly negatively correlated with B cell (*p* < 0.001), CD8^+^ T cell (*p* < 0.001), CD4^+^ T cell (*p* < 0.001), macrophage (*p* < 0.05), neutrophil (*p* < 0.001), and dendritic cell infiltration (*p* < 0.001) (Figure 5B).

At the same time, it is considered that SEC61G may participate in endopeptidase activity and MHC class I protein complex binding in GO analyses. Besides, antigen peptide transporter (TAP) is to transport the peptide to the endoplasmic reticulum, and subsequent peptide loading by MHC class I molecules. So, we analyzed the association between TAP1 and TAP2 expression with SEC61G. Results showed that TAP 1 and TAP2 expression was negatively correlated with SEC61G expression in the HNSCC‐TCGA datasets (Figure 5C,D).

### 3.6 SEC61G predicts the efficacy of immune checkpoint inhibitors

PD‐L1 (CD274) expression plays a vital role in tumor immune escape and is also a predictive marker for therapeutic efficacy of immune checkpoint inhibitors (ICIs). The result showed that PD‐L1 expression was also negatively correlated with SEC61G expression in the HNSCC‐TCGA datasets (Figure 5E). Considering that SEC61G was negatively correlated with immune cell infiltration and PD‐L1 expression, we analyzed SEC61G expression during immune checkpoint therapy. A dataset (IMvigor 210 data) generated from metastatic urothelial cancer patients treated with atezolizumab was downloaded. Kaplan–Meier survival analysis showed that patients with high SEC61G expression had a lower OS than patients with low‐SEC61G expression (*p* = 0.006, Breslow test) (Figure 5F).

### 3.7 SEC61G is upregulated and correlated with adverse outcome in oropharyngeal cancer

**FIGURE 6:**
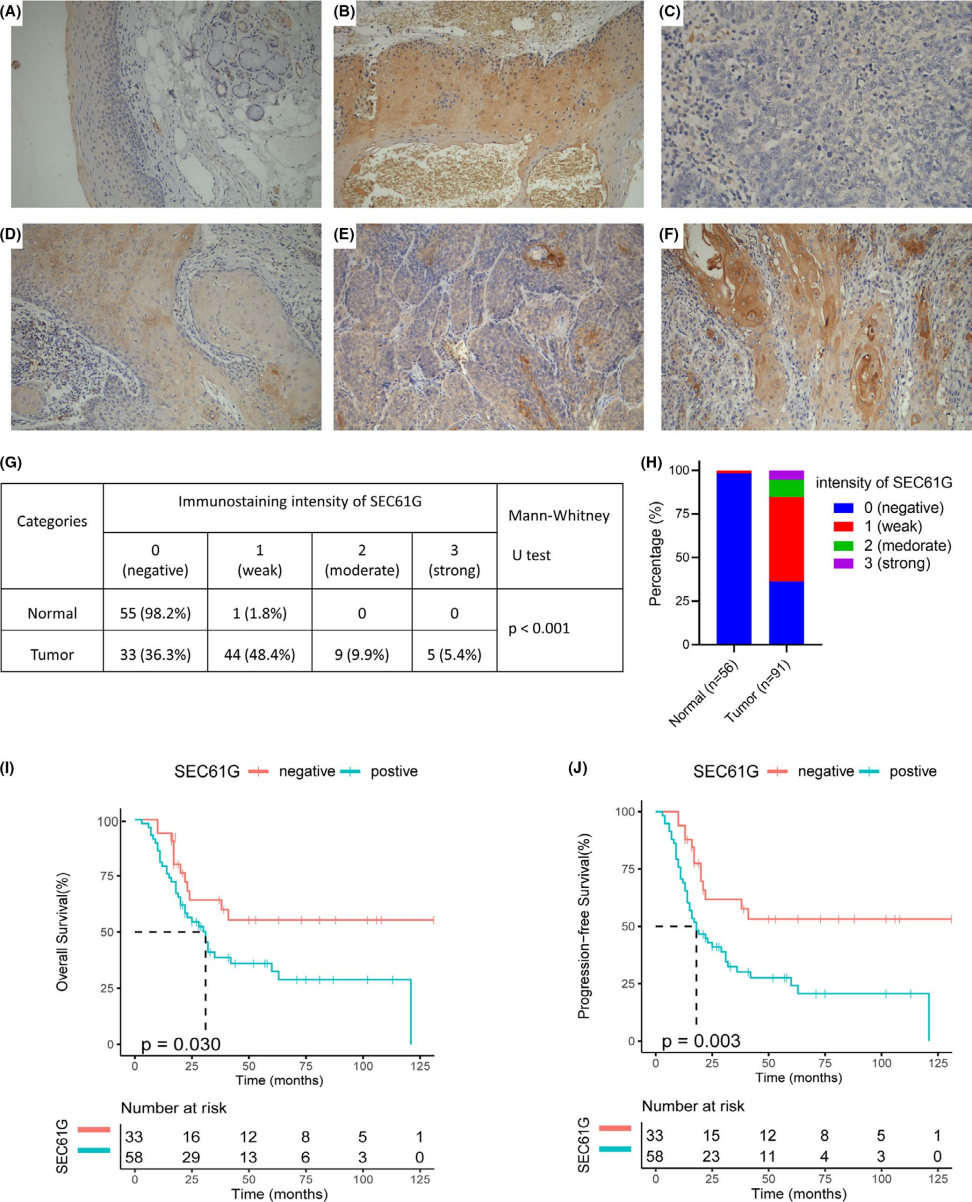
The expression and prognostic values of SEC61G in oropharyngeal cancer: (A, B) representative IHC staining intensities of SEC61G in normal tissues: SEC61G was scored as (A) negative and (B) weak; (C–F) representative IHC staining patterns of SEC61G in OPC tissues: (C) negative, (D) weak, (E) moderate and (F) strong; (G, H) the proportions of SEC61G in normal and tumor tissues; (I) Overall survival rate of SEC61G in oropharyngeal cancer; (J) Progression‐free survival of SEC61G in oropharyngeal cancer

To verify the difference of SEC61G expression and its prognostic value in HNSCC, we used 91 OPC cases and 56 normal adjacent tissue samples from our center to detect SEC61G protein expression by IHC. According to the staining intensity, SEC61G expression was divided into negative (Figure 6A,C), weakly (Figure 6B,D), moderately (Figure 6E), and strongly positive (Figure 6F), as shown in Figure 6. Among the normal tissue samples, 55 cases (98.2%) were negative, and only 1 case (1.8%) was weakly positive. Among the OPC 91 cases, 44 cases were weakly positive, 9 were moderately positive, and 5 were strongly positive. The overall SEC61G‐positive rate was 63.7%, and only 33 cases (36.3%) were SEC61G‐negative (Figure 6G). Rank‐sum tests showed that SEC61G expression in OPC tissues was higher than expression in normal adjacent tissues (*p* < 0.001, Figure 6H). The median follow‐up time was 60 months (range 3–131 months). Detailed demographic and clinical characteristics are listed in Table 3. Based on SEC61G expression in the tumor tissues, OPC patients were divided into negative and positive expression groups. SEC61G expression correlated to the degree of pathological keratinization, that is, the proportion of SEC61G‐positive samples patients with keratinization was higher than in patients with non‐keratinization (*p* = 0.009) (Table 3). Kaplan–Meier survival analyses showed that patients with high SEC61G expression had a lower OS (55.4% vs. 32.4%, *p* = 0.030) and PFS (53.2% vs. 24.1%, *p* = 0.003) than did patients with low‐SEC61G expression (Figure 6I,J). MAV confirmed that high SEC61G expression was an independent inferior factor for OS (HR = 2.46, 95% CI: 1.15–5.28, *p* = 0.021) and PFS (HR = 2.82, 95% CI: 1.36–5.85, *p* = 0.005) when adjusted for sex, age, clinical stage, and chemotherapy cycles (Table 4).

**TABLE 3:** Demographic and clinical characteristics of oropharyngeal cancer patients with negative and positive SEC61G

Categories	Level	SEC61G expression		
		Negative (*n* = 33)	Positive (*n* = 58)	*p*‐value
Gender	Female	6	5	0.197
	Male	27	53	
Age (year)	<60	18	34	0.706
	≥60	15	24	
T classification	T1–T2	18	29	0.737
	T3–T4	15	28	
N classification	N0–1	11	15	0.448
	N2–3	22	43	
Clinical stage	I–II	4	16	0.087
	III–IV	29	42	
Smoking	No	11	22	0.513
	Yes	21	31	
Differentiated	Poor	9	8	0.193
	Moderately	17	30	
	Well	7	20	
Keratinization	No	14	10	0.009
	Yes	19	48	
Treatment	RT ± CT	21	31	0.345
	Surgery ± RT ± CT	12	27	

Abbreviations: CT, chemotherapy; RT, radiotherapy.

**TABLE 4:** The multivariate analysis of overall survival and progression‐free survival according to SEC61G expression, after adjusting for other potential predictors in oropharyngeal cancer

Characteristics	OS		PFS	
	HR (95% CI)	*p* value	HR (95% CI)	*p* value
Gender (Female vs. Male)	0.83 (0.22–3.16)	0.780	0.85 (0.23–3.12)	0.808
Age (≤60 vs. >60)	2.52 (1.27–5.00)	0.008	2.16 (1.14–4.09)	0.018
Clinical stage (Ⅰ–Ⅱ vs. Ⅲ–Ⅳ)	4.02 (1.37–11.83)	0.012	2.86 (1.17–7.00)	0.021
Smoking (No vs. Yes)	1.67 (0.70–3.975)	0.243	1.67 (0.74–3.77)	0.213
Treatment (RT ± CT vs. Surgery ± RT ± CT)	0.46 (0.23–3.91)	0.025	0.57 (0.30–1.08)	0.085
Differentiated		0.848		0.348
(Poor vs. moderately)	0.75 (0.23–2.49)	0.637	0.48 (0.17–1.35)	0.165
(Poor vs. well)	0.87 (0.25–3.09)	0.831	0.46 (0.15–1.39)	0.171
Keratinization (No vs. yes)	2.91 (0.77–11.03)	0.105	3.20 (0.98–10.48)	0.054
SEC61G (Negative vs. positive)	2.46 (1.15–5.28)	0.021	2.82 (1.36–5.85)	0.005
P16 (Negative vs. positive)	0.92 (0.29–2.91)	0.886	1.02 (0.34–3.03)	0.971

Abbreviations: CT, chemotherapy; OS, overall survival; PFS, progression‐free survival; RT, radiotherapy.

### 3.8 The expression and prognostic value of the other subunits of the SEC61 complex

**FIGURE 7:**
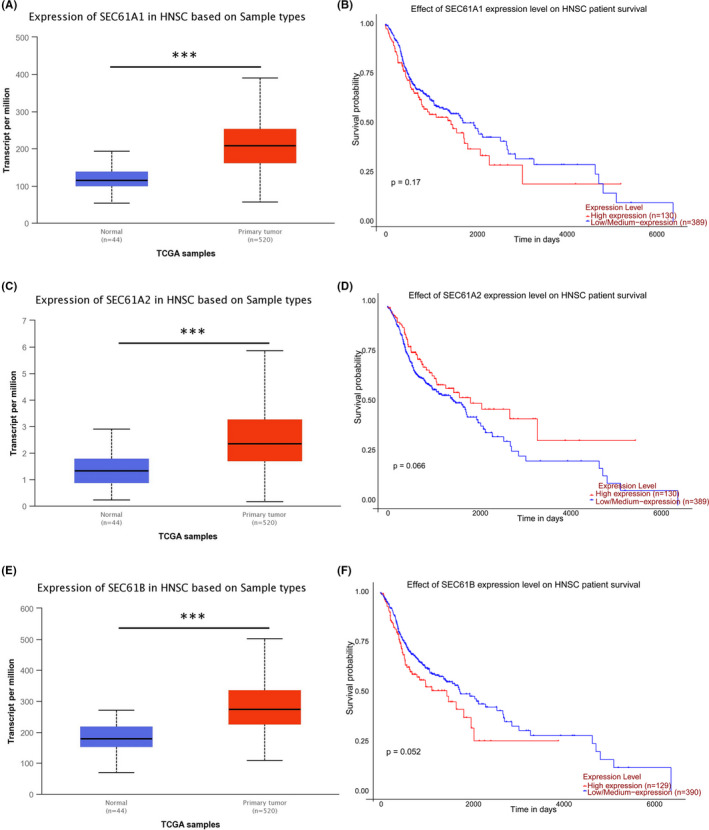
The expression and prognostic value of the other subunits of the SEC61 complex in HNSCC: (A) SEC61A1 expression levels in HNSCC in TCGA; (B) Overall survival curve of SEC61A1 in TCGA‐HNSCC; (C) SEC61A2 expression levels in HNSCC in TCGA; (D) Overall survival curve of SEC61A2 in TCGA‐HNSCC; (E) SEC61B expression levels in HNSCC in TCGA; (F) Overall survival curve of SEC61B in TCGA‐HNSCC. (*p < 0.05, **p < 0.01, ***p < 0.001)

The complex has three subunits, namely α, β, and γ. The expression and prognostic value of SEC61G have been effectively analyzed and verified. We also tried to analyze other subunits of SEC61. The results showed that the expressions of SEC61A1, A2, and B in HNSCC were higher than those in normal control tissues by UALCAN online tool, respectively (Figure 7A, *p* < 0.001; Figure 7C, *p* < 0.001; and Figure 7E, *p* < 0.001). However, Kaplan–Meier survival analysis showed that these subunits did not significantly affect the prognosis of HNSCC in TCGA data (Figure 7B, *p* = 0.17; Figure 7D, *p* = 0.066; and Figure 7F, *p* = 0.052).

## 4 DISCUSSION

HNSCC is a group of heterogeneous cancers originating from epithelial cells of the head and neck.^26^ The clinical HNSCC outcomes are far from satisfactory using current treatments. Therefore, it is essential to find stable broad‐spectrum biomarkers to predict prognosis and guide individualized treatments. By analyzing Oncomine and TCGA datasets, we found SEC61G was upregulated in HNSCC and correlated with inferior OS. Meanwhile, the prognostic value of SEC61G was validated using a GEO dataset (GSE65858). SEC61G CNV DNA amplification (9.66%) was associated with high SEC61G expression and correlated with lower OS in HNSCC. Functional enrichment analyses found that SEC61G expression and DNA amplification were associated with immune response and protein metabolism. Further analyses showed that SEC61G overexpression and DNA amplification were negatively associated with immune cell infiltration. Further, SEC61G expression negatively correlated with TAP1, TAP2, and PD‐L1 expression, and indicated a lower median OS in patients receiving ICIs. Furthermore, we confirmed that SEC61G was highly expressed in HNSCC using IHC and is an independent prognostic factor for OPC. Thus, our study provides new insights into understanding the potential roles of SEC61G in tumor immunology and its potential use as a cancer biomarker.

In this study, we show that SEC61G is highly expressed in various tumors using Oncomine and TCGA datasets. Further, these datasets showed that SEC61G was highly expressed in HNSCC, regardless of HPV infection. Similarly, high SEC61G expression in HNSCC was confirmed using a GEO dataset. SEC61G protein was also highly expressed in OPC, while the normal group had little SEC61G. In HNSCC, OPC is a relatively unique tumor, and its occurrence and development are closely related to HPV infection. In recent years, studies have also found that HPV DNA detection can be used as an early diagnosis method for HPV‐related OPC.^27^, ^28^ Although SEC61G expression in tumors is rarely reported, two studies found that SEC61G is highly expressed in gastric cancer^16^ and glioblastoma.^15^ These findings were consistent with our findings. Importantly, our results indicate that SEC61G is highly expressed in HNSCC, that patients with high SEC61G expression have a worse OS, and that SEC61G is an independent prognostic factor of OS. We also found that the OS and PFS of SEC61G‐positive patients were significantly worse than in SEC61G‐negative patients. Multivariate analysis confirmed that the SEC61G protein level is another independent prognostic factor of OS and PFS in OPC. Further, the prognostic value of SEC61G in HNSCC is not affected by HPV status, that is, high SEC61G expression indicates poor prognosis in both HPV‐positive and ‐negative HNSCC. A recent study also demonstrated that high SEC61G expression is associated with worse OS in glioblastoma,^15^ which was consistent with our study. It is interesting that the subunits of SEC61 are highly expressed in HNSCC, but other subunits except SEC61G are not prognostic factors for HNSCC. This indicates that SEC61G plays an extremely important role in HNSCC. It is worth noting that SEC62, an interaction partner of SEC61, is overexpressed and associated with a poor prognosis of HNSCC.^29^ This indicates that the SEC61 family and its partners play an extremely important role in HNSCC.

Although there are many reasons contributing to increased gene expression, DNA methylation and CNVs are the most common. DNA methylation is a common epigenetic mechanism present in all forms of cancer.^30^ Promoter methylation accompanies gene silencing.^31^ In this study, further analyses showed that SEC61G expression is negatively associated with methylation, while SEC61G promoter methylation in HNSCC is lower than in normal tissue. SEC61G demethylation might partly contribute to SEC61G upregulation in HNSCC, but this hypothesis remains to be validated. Somatic mutation is a hallmark of cancers, including DNA amplification.^32^ In HNSCC, 9.66% of patients were found to have SEC61G DNA amplification. More importantly, our study found that patients with SEC61G DNA amplification had a worse OS than patients without amplification. This DNA amplification might also partly explain the SEC61G overexpression we observed in HNSCC. Jim Sheu et al. reported that DNA amplification of SEC61G was not significantly increased, and SEC61G mRNA did not increase significantly.^33^ They believed that SEC61G might be a passenger gene in HNSCC rather than a driver gene. Our results are not completely consistent with their results, which may be caused by the larger sample size and the different detection methods in our study. It was worth noting that our data found that the expression of SEC61G was strong correlation to the state of keratinization. It could be a potential mechanism of how SEC61G is contributing to cancer prognosis. This phenomenon needs more data and mechanism research to confirm.

SEC61G is a subunit of the SEC61 complex, which is a central component of the protein translocation apparatus in the endoplasmic reticulum membrane.^13^, ^14^ At present, the biological functions of SEC61G in tumors are only partially understood. Studies from non‐small cell lung cancer and glioma indicate that SEC61G promotes tumor proliferation.^17^, ^25^ However, it is worth noting that our GSEA and GEO biological function analyses showed that SEC61G was not significantly enriched in proliferation‐associated terms but was enriched with immune response, T‐cell receptor, B‐cell receptor, chemokine signaling pathway, and leukocyte trans‐endothelial migration. In addition, TIMER analyses showed that SEC61G expression and DNA amplification were negatively correlated with immune cell infiltration, including dendritic cells, CD4+ T cells, CD8+ T cells, and B cells. Further, GSEA and GO analyses showed that SEC61G might be involved in protein degradation and transport, MHC protein binding, and MHC class I protein complex binding. Interestingly, we found that SEC61G expression was negatively correlated with TAP1 and TAP2 expression. Previous studies showed that SEC61G participates in forming transmembrane pores and mediates nascent polypeptide degradation.^13^, ^14^ Thus, we hypothesize that SEC61G might mediate tumor antigen degradation and reduce the formation of MHC class I molecules. Without MHC class I molecules, dendritic cells cannot effectively recognize and present tumor antigens, leading to inefficient immune cell recruitment and activation, especially for cytotoxic T cells.^34^ This evidence is consistent with the negative correlation between SEC61G expression and immune cell infiltration in HNSCC. Interestingly, SEC61G expression was negatively correlated with PD‐L1 expression in HNSCC, and patients with high SEC61G expression seemed to have lower OS than did patients with low‐SEC61G expression in the dataset of metastatic urothelial cancer patients treated with atezolizumab. These findings suggest that SEC61G may affect immune cell infiltration and immunotherapy efficacy, which makes it a predictive biomarker for immunotherapy. These results also suggest that SEC61G is a potential therapeutic target that may promote immune cell infiltration and enhance the therapeutic effect of ICIs.

Although this study improved our understanding of SEC61G in HNSCC, there were some limitations. First, the detailed mechanisms of the SEC61G‐mediated immune escape are unclear. While we found that SEC61G is negatively correlated with immune cell infiltration in the tumor microenvironment, the conclusion that SEC61G promotes antigen degradation and decreases the expression of antigen presentation‐related proteins is based only on bioinformatics analysis. Although there was a certain correlation between SEC61G expression and TAP1 and TAP2, these correlations were quite weak, and they cannot be ruled out that they were due to the large sample size. In vivo and in vitro experiments are needed to verify this conclusion. Thus, we will continue to explore the mechanism of SEC61G‐mediated immune escape in a future study. Second, the observation that SEC61G may be a potential efficacy marker for immune checkpoint inhibitor treatment lacks supporting clinical data. The main reason is that few studies on SEC61G are currently done, and SEC61G is not included in the commonly used gene sequencing panel, which limits the availability of data to verify the value of SEC61G in ICIs treatment.

## 5 CONCLUSIONS

Our findings suggest that SEC61G overexpression is an independent adverse prognostic factor in HNSCC. Promoter demethylation and DNA amplification might contribute to SEC61G upregulation, and SEC61G DNA amplification is associated with poor outcome. SEC61G mediates reduced immune cell infiltration in the tumor microenvironment. This study demonstrates SEC61G as a prognostic biomarker for HNSCC, highlighting its potential as a predictive biomarker and a therapeutic target.

## CONFLICT OF INTEREST

The authors declare that the research was conducted in the absence of any commercial or financial relationships that could be construed as a potential conflict of interest.

## ETHICAL APPROVAL STATEMENT

This study was approved by the Hospital Review Board of Fujian Cancer Hospital, Fujian, China.

## Data Availability

The datasets used and/or analyzed during the current study are available from the corresponding author upon reasonable request.
